# Cervical and Cesarean Scar Pregnancy in One Patient: A Sequential Case with Literature Review

**DOI:** 10.3390/jcm15103949

**Published:** 2026-05-20

**Authors:** Zofia Malczewska, Agata Chojnicka, Łucja Zaborowska, Artur Ludwin

**Affiliations:** 1st Department of Obstetrics and Gynecology, Medical University of Warsaw, pl. Starynkiewicza 1/3, 02-015 Warsaw, Poland

**Keywords:** cervical pregnancy, cesarean scar pregnancy, non-tubal ectopic pregnancy, hysteroscopy, fertility-preserving treatment, transvaginal ultrasound

## Abstract

**Background**: Cervical pregnancy and cesarean scar pregnancy are rare forms of non-tubal ectopic pregnancy associated with a high risk of severe hemorrhage, surgical intervention, and potential loss of fertility. We describe a unique case of sequential abnormal implantation in which a cervical pregnancy was followed by a cesarean scar pregnancy one year later. The occurrence of two distinct forms of non-tubal ectopic pregnancy in a single patient represents an exceptionally uncommon clinical scenario, underscoring the importance of early diagnosis and carefully planned treatment. **Case presentation**: A 39-year-old woman, gravida 4 para 3, was diagnosed with two distinct non-tubal ectopic pregnancies over a 1-year period. The first pregnancy was implanted in the cervical canal, whereas the second was located within the cesarean section scar. In each episode, the diagnosis was established early by transvaginal ultrasound. As the patient was hemodynamically stable and wished to preserve fertility, minimally invasive hysteroscopic evacuation was performed in both pregnancies. The procedures were completed without significant intraoperative bleeding, and no additional hemostatic interventions were required. Follow-up serum β-hCG levels became negative after treatment, confirming complete resolution of pregnancies. **Conclusions:** This case demonstrates that early ultrasonographic diagnosis and careful individualized management may enable successful fertility-preserving treatment even in exceptionally rare cases. It also supports the potential role of minimally invasive approaches in selected hemodynamically stable patients and highlights the need for standardized management protocols for cervical and cesarean scar pregnancy.

## 1. Introduction

Ectopic pregnancy (EP) is defined as an implantation of a fertilized egg outside of the endometrial cavity of the uterus [1]. The majority of ectopic pregnancies occur within the fallopian tube, most commonly in the tubal ampulla, representing approximately 95% of cases. However, implantation may also occur in other, less common locations, such as the interstitial portion of the fallopian tube, cervical canal, ovary, cesarean scar defect and abdominal cavity [2] (Table 1).

Considering the potentially severe complications of EP and the significant maternal morbidity and mortality associated with this condition, identification of predisposing factors is crucial. Recognition of high-risk groups allows for closer surveillance, earlier diagnosis, improved clinical outcomes and the development of preventive strategies. Risk factors of EP include a broad spectrum of clinical, reproductive and behavioral determinants that predispose to abnormal implantation outside of the uterine cavity. A history of prior ectopic pregnancy represents one of the strongest predictors of recurrence, likely reflecting persistent tubal pathology or residual structural damage [3]. Pelvic inflammatory disease and previous sexually transmitted infections increase risk through inflammation-induced scarring and tubal ciliary dysfunction [4]. Infertility and the use of assisted reproductive technologies (ART) are also associated with elevated EP rates [5]. Anatomical alterations following uterine or tubal procedures may also interfere with physiological embryo transport, increasing the likelihood of ectopic pregnancy. Additional factors include: advanced maternal age, cigarette smoking, presence of intrauterine device (IUD) and a history of spontaneous or induced abortion [6,7]. It is important to emphasize that EP may occur in the absence of identifiable risk factors, underscoring the need for sustained clinical vigilance in all early pregnancies presenting with abdominal pain or vaginal bleeding, which represent the most common clinical manifestations of this condition [1]. Delayed or missed diagnosis may lead to severe and life-threatening complications. Progressive invasion of trophoblastic tissue can result in rupture of the implantation site, causing massive hemorrhage and acute hemodynamic instability. Ruptured EP is the leading cause of maternal death in the first trimester, accounting for approximately 9–13% of pregnancy-related deaths worldwide [4].

Upon confirmation of ectopic pregnancy, therapeutic management may involve pharmacologic, surgical, or expectant interventions, with modern practice prioritizing fertility-sparing approaches whenever possible [3].

This report presents a unique case of sequential abnormal implantation, in which a cervical pregnancy (CP) was followed by cesarean scar pregnancy (CSP) one year later in the same patient. The occurrence of two distinct forms of non-tubal ectopic implantation represents an exceptionally uncommon clinical scenario, underscoring the importance of early diagnosis and structured treatment strategies.

## 2. Case Presentation

### 2.1. Cervical Pregnancy

**Figure 1:**
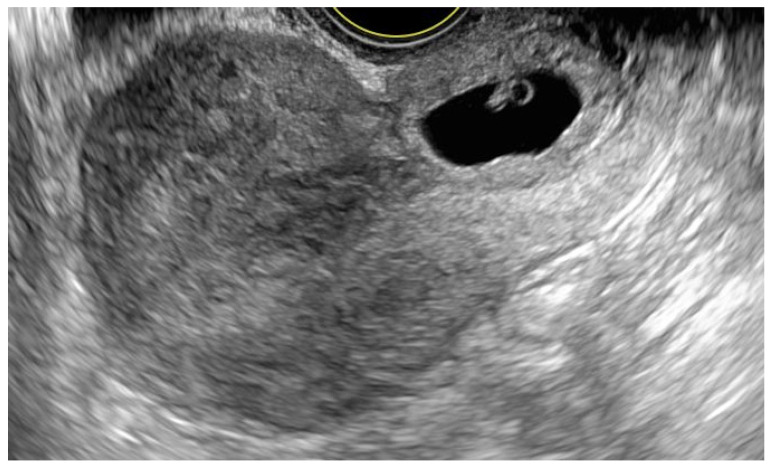
Two-dimensional transvaginal ultrasound image in sagittal plane demonstrating a gestational sac located within cervical canal.

A 39-year-old Caucasian woman, gravida 4, para 3, was referred to the 1st Department of Obstetrics and Gynecology of Warsaw Medical University due to a suspicion of an ectopic pregnancy. According to her last menstrual period, the gestational age was 6 weeks and five days. Her medical history included Strassman metroplasty via laparotomy for a bicornuate uterus and diagnostic laparoscopy, both performed 17 years earlier, as well as three cesarean sections performed 5, 12, and 16 years earlier. She was a long-term smoker and continued smoking cigarettes during this pregnancy. The patient’s vital signs were stable, and there was no sign of pelvic pain or vaginal bleeding during physical examination. The initial β-hCG level was 16 818 mIU/mL. The transvaginal ultrasound revealed an empty uterine cavity and a gestational sac measuring 17.8 × 5.4 mm containing an embryo with cardiac activity located in the cervical canal (Figure 1).

**Figure 2:**
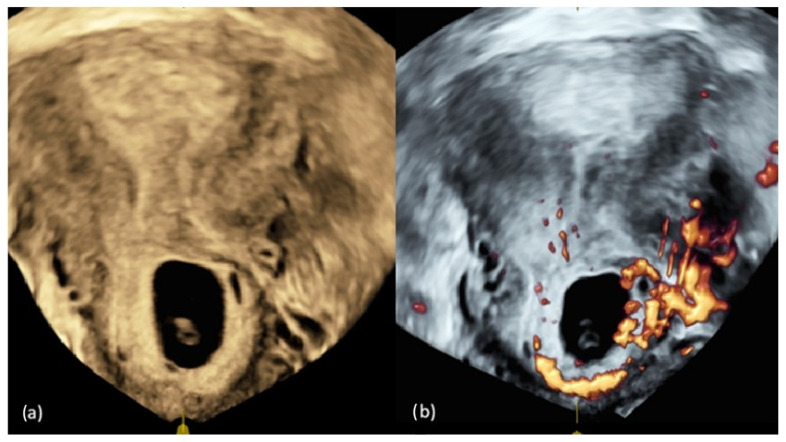
(**a**) Three-dimensional transvaginal ultrasound image obtained at the initial diagnostic stage in a patient with a viable cervical pregnancy at approximately 6 weeks of gestation. The coronal plane shows the gestational sac implanted within the cervical canal; (**b**) Power Doppler ultrasound of the same view demonstrating marked peritrophoblastic vascularization. Doppler imaging was performed to assess the risk of hemorrhage associated with the abnormal implantation site.

The measured crown-rump length was 3.7 mm, corresponding to a gestational age of 5 weeks and 5 days (Figure 2a). Transvaginal ultrasound with color Doppler revealed significant vascularity in the cervical region, consistent with an increased risk of hemorrhage (Figure 2b).

Despite this, and in the context of a hemodynamically stable patient with no active bleeding, we decided to initiate systemic methotrexate therapy as a first-line approach, taking into account the patient’s preference to preserve her uterus and avoid definitive loss of reproductive potential, even though future childbearing was not her primary goal.

The patient was thoroughly informed regarding all available treatment options, including systemic and local pharmacotherapy, hysteroscopic and surgical management, and uterine artery embolization (UAE). The potential benefits, risks, and likelihood of success associated with each approach were discussed in detail, including the possibility of treatment failure, need for additional interventions, and the risk of hemorrhagic complications. This shared decision-making process led to the selection of conservative management aimed at preserving uterine integrity while maintaining patient safety. UAE, although effective in hemorrhage control, was not chosen at this stage, as it is associated with potential complications such as post-embolization syndrome, pelvic infection, endometritis, ovarian dysfunction, reduced ovarian reserve, intrauterine adhesions, uterine ischemia or necrosis, thromboembolic events, and, rarely, hysterectomy. Fertility-related consequences such as premature ovarian insufficiency and infertility have also been reported in selected patients [8,9].

After confirming normal liver and renal function, systemic methotrexate was administered intramuscularly at a dose of 92 mg (50 mg/m^2^ of body surface area). On the first day following methotrexate administration, vaginal spotting was observed during speculum examination. On Day 3, serum β-hCG level continued to rise, and embryonic cardiac activity persisted. Consequently, a second dose of methotrexate (92 mg) was administered. Folate supplementation was provided orally. Due to a significant increase in liver enzyme levels—ALT rising to 252 U/L and AST reaching 109 U/L—further doses of methotrexate were withheld. A normalization of liver enzyme levels was observed by the 10th day of hospitalization. The patient reported lower abdominal pain, which subsided within two days without additional treatment. On Day 7, we observed the cessation of embryonic cardiac activity and a downward trend in β-hCG levels (Table 2).

**Figure 3:**
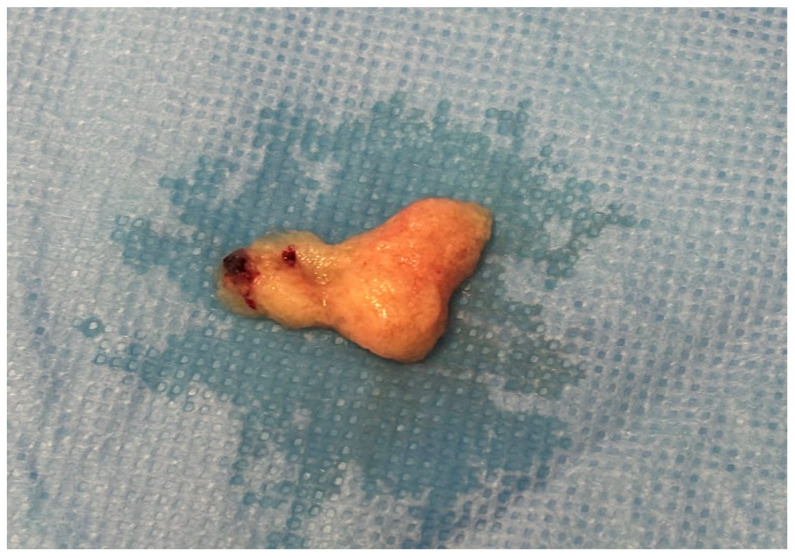
Gestational sac of a cervical pregnancy, removed intact using the “cold loop” technique on day 11 following the initiation of systemic methotrexate therapy (two doses). At the time of the procedure, the pregnancy was non-viable. The gestational age, calculated from the last menstrual period, was 7 weeks and 1 day. The sac was mechanically enucleated without electrosurgical energy, after prior cervical dilation and under transrectal ultrasound guidance. The image shows the fully extracted gestational sac, confirming complete and atraumatic removal.

On the 11th day of hospitalization, under general anesthesia, a hysteroscopic evacuation of the cervical pregnancy was performed after β-hCG decreased from 29,600 mIU/mL to 17,126 mIU/mL, indicating trophoblastic regression and allowing safer surgical management. Prior to the insertion of the hysteroscope, the cervical canal was carefully dilated to facilitate access. A bipolar resectoscope with a loop electrode was then introduced, allowing direct visualization of the pregnancy implanted within the cervical canal. The loop electrode was used solely as a mechanical instrument, without activation of electrical current. The gestational sac was enucleated in its entirety using the “cold loop” technique, with gentle mechanical maneuvers and medium-pressure distension lavage to separate the chorionic villi from the cervical mucosa (Figure 3).

**Figure 4:**
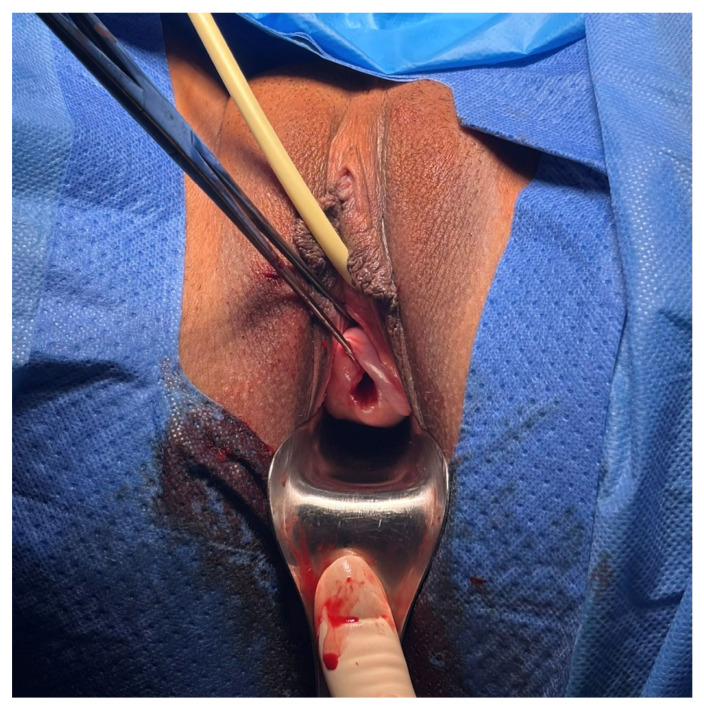
Postoperative speculum view of the cervix following cold loop enucleation of the gestational sac. No active bleeding was observed, and additional hemostatic measures, such as balloon tamponade, were not required.

The procedure was performed under transrectal ultrasound guidance, which provided stable real-time visualization during cervical instrumentation while avoiding contamination of the sterile operative field by a transvaginal probe. The procedure was uncomplicated with no ongoing hemorrhage (Figure 4).

Additional hemostatic measures, such as balloon tamponade, were not required. The patient was discharged home on the first day following the surgery. Only vaginal spotting was observed during the first week following surgery. Histopathological examination confirmed products of conception, including trophoblastic tissue, decidual fragments, and secretory endometrium. No features of gestational trophoblastic disease were identified.

**Figure 5:**
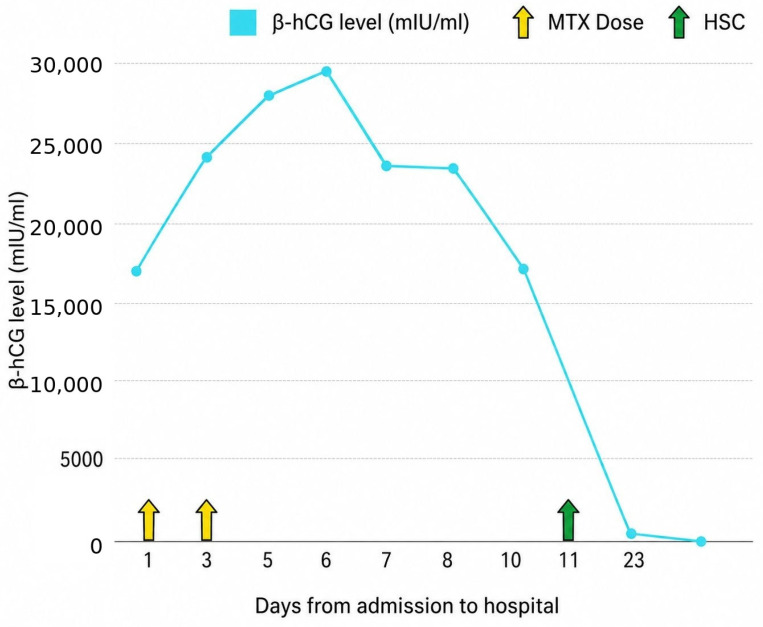
Evolution of serum β-hCG during methotrexate treatment of cervical pregnancy. An initial rise in β-hCG after MTX administration was observed, which may occur during early response to treatment and should not be interpreted in isolation as treatment failure. Subsequent decline in β-hCG, together with cessation of embryonic cardiac activity and reduced trophoblastic vascularity, supported the decision to proceed with delayed hysteroscopic evacuation. MTX—methotrexate; HSC—hysteroscopy.

On the 23rd day of the hospitalization, the β-hCG level decreased to 65.74 mIU/mL and liver enzyme levels were normal (Figure 5).

**Figure 6:**
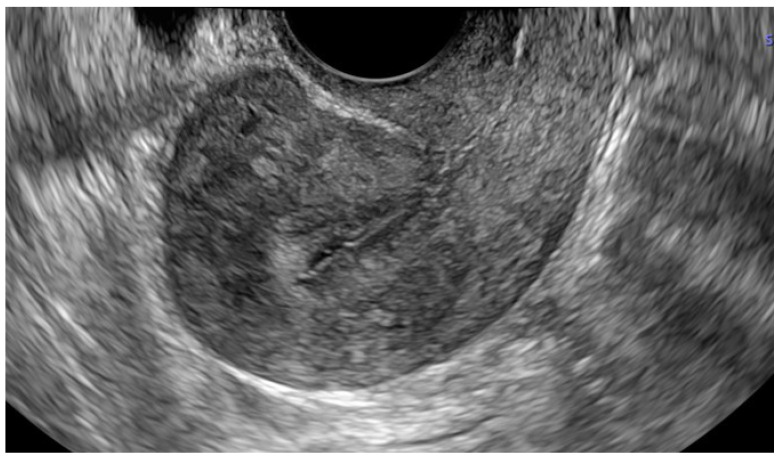
Two-dimensional transvaginal ultrasound image obtained six months after treatment, demonstrating normal cervical and uterine anatomy, with no pathological findings.

The patient did not attend any further in-person follow-up visits. She was last contacted by telephone approximately five weeks after the procedure and reported feeling well, with no symptoms. She declined additional outpatient visits due to the long travel distance and did not obtain any further β-hCG testing. However, approximately six months after treatment, the patient returned for an in-person follow-up. Serum β-hCG was confirmed to be negative, and transvaginal ultrasound demonstrated normal uterine and cervical anatomy without any abnormalities (Figure 6). Throughout the entire treatment course, there were no instances of significant vaginal bleeding or abdominal discomfort.

### 2.2. Cesarean Scar Pregnancy

**Figure 7:**
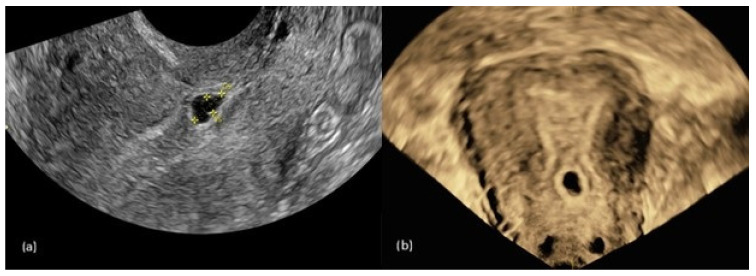
(**a**) Two-dimensional transvaginal ultrasound image and (**b**) three-dimensional transvaginal ultrasound image showing a gestational sac located in the anterior lower uterine segment at the site of the previous cesarean scar.

One year later, the patient presented to the outpatient clinic with suspicion of pregnancy due to a missed menstrual period. Serum β-human chorionic gonadotropin was 2279 mIU/mL. Transvaginal ultrasonography revealed a gestational sac measuring 7.3 mm in diameter with a yolk sac measuring 3.2 mm and a subchorionic hematoma (Figure 7). Fetal cardiac activity was not detected. The estimated gestational age was 5 weeks and 3 days.

The patient denied abdominal pain but reported moderate vaginal bleeding. On physical examination, only light vaginal spotting was observed. Given the presence of a large subchorionic hematoma and accompanying symptoms, spontaneous miscarriage was considered a possible clinical outcome. As the patient remained hemodynamically stable, short-term follow-up was recommended, with reassessment scheduled four days later. At this follow-up visit, serum β-hCG had increased from 2279 to 3919 mIU/mL. The patient was admitted to the hospital on the same day, and repeat transvaginal ultrasonography performed on admission showed no significant change compared with the previous examination. Hysteroscopic evacuation was scheduled for the following day. The procedure was performed under general anesthesia. After gentle cervical dilation, the uterine cavity was accessed with a bipolar resectoscope equipped with a loop electrode. Hysteroscopic inspection demonstrated trophoblastic tissue implanted in the anterior lower uterine segment, corresponding to the previous cesarean section scar. The products of conception were removed completely using a cold-loop technique. The loop electrode was used solely as a mechanical instrument, without activation of electrical current. Controlled irrigation and careful blunt dissection were used to detach the chorionic villi from the surrounding myometrial tissue.

The procedure was completed without intraoperative complications, and no active bleeding was observed. Additional hemostatic interventions were not required. Histopathological examination demonstrated decidual tissue with focal hemorrhagic necrosis and chorionic villi with degenerative changes, consistent with products of conception. The patient was discharged home on the following day in good clinical condition. During the subsequent month, she did not report any concerning symptoms. Follow-up serum β-hCG testing performed approximately 1 month after the procedure was negative, confirming complete resolution of the pregnancy.

## 3. Literature Review

### 3.1. Methodology

We performed a structured narrative review to provide clinical context for the presented sequential case and to summarize evidence regarding risk factors, diagnostic considerations, fertility-preserving treatment strategies, and reported reproductive outcomes in cervical pregnancy and cesarean scar pregnancy. Although not conducted as a systematic review, the search and selection process was designed to be transparent and reproducible. PubMed/MEDLINE and Google Scholar were searched up to March 2026 using combinations of the terms “cervical pregnancy”, “cervical ectopic pregnancy”, “cesarean scar pregnancy”, “non-tubal ectopic pregnancy”, “methotrexate”, “hysteroscopy”, “dilatation and curettage”, “uterine artery embolization,” “fertility-preserving treatment,” “conservative management”, “treatment outcome” and “reproductive outcome.” Reference lists of relevant articles and reviews were manually screened. Eligible publications included systematic and scoping reviews, clinical guidelines or expert recommendations, cohort studies, case series, and clinically relevant case reports reporting diagnosis, management, complications, treatment success, or reproductive outcomes in CP or CSP. When multiple publications addressed the same treatment modality, priority was given to recent studies, systematic or scoping reviews and articles published in peer-reviewed journals with higher scientific impact or greater relevance to obstetrics and gynecology. Publications were excluded if they did not address CP or CSP, did not provide extractable treatment or outcome data, were unavailable in full text, represented duplicate reports, or were published in languages other than English.

### 3.2. Risk Factors

Non-tubal ectopic pregnancies are strongly associated with factors that disrupt the integrity of the endometrial-myometrial interface, also known as “junctional zone”, and promote implantation within abnormal tissue planes. Prior uterine and cervical instrumentation have been consistently reported among the most important risk factors for cervical implantation and cesarean scar pregnancy, likely as a result of structural alterations of the uterine wall and disruption of the inner myometrium. CSP is increasingly recognized as an iatrogenic consequence of previous cesarean delivery, with implantation occurring within a scar “niche” of the anterior lower uterine segment. In addition, cigarette smoking is a well-established independent risk factor for ectopic pregnancy. Tobacco exposure is thought to impair endometrial receptivity and alter local vascularization, potentially affecting the structural and functional integrity of uterine and cervical tissues and thereby increasing susceptibility to abnormal implantation. Notably, a history of previous ectopic pregnancy itself constitutes a significant risk factor for recurrence, with studies indicating a substantially increased likelihood of subsequent abnormal implantation [6,7]. In the present case, the patient belongs to a clearly high-risk group due to active smoking and a history of multiple uterine surgeries, including metroplasty and three cesarean deliveries, all of which may have contributed to alterations in the uterine architecture and predisposed the patient to sequential abnormal implantation at distinct non-tubal sites. Therefore, careful consideration of individual risk factors is essential when selecting a treatment strategy and anticipating the potential need for a combined or stepwise therapeutic approach. The management of non-tubal ectopic pregnancies remains a significant challenge, with treatment strategies tailored to individual cases based on hemodynamic stability, gestational age, β-hCG levels and fertility preservation desires [2].

### 3.3. Cervical Pregnancy Management

The management of cervical pregnancy (CP) remains challenging, and several medical and surgical treatment options have been described in the literature (Table 3).

Among the most commonly employed therapeutic methods is systemic or intra-amniotic administration of methotrexate (MTX), which effectively inhibits trophoblastic proliferation [1]. MTX is used in single or multiple doses intramuscularly or injected directly into the gestational sac under ultrasound guidance [26]. Intra-amniotic injection has also been combined with agents such as potassium chloride (KCl), absolute ethanol, prostaglandins, hyperosmolar glucose or vasopressin to induce fetal demise or reduce vascularization at the implantation site. These combined approaches may enhance treatment efficacy while minimizing the risk of hemorrhage [27,28,29]. In particular, intra-amniotic administration of KCl or ethanol has shown promising results, especially in cases where systemic MTX is ineffective or contraindicated [30,31,32]. However, the effectiveness of primary MTX therapy appears to be lower in more advanced cases of CP. Several factors have been associated with a higher risk of unsuccessful MTX treatment, including β-hCG > 10,000 mIU/mL, gestational age > 9 weeks, the presence of embryonic cardiac activity, and CRL > 10 mm [30,33]. An important consideration in the management of CP is the identification of contraindications to MTX therapy. Absolute contraindications include chronic liver or renal disease, hematologic disorders, immunodeficiency, active pulmonary disease, peptic ulcer disease, and known hypersensitivity to MTX [1,34]. Relative contraindications are high initial β-hCG levels, large gestational sac size, and the presence of embryonic cardiac activity, which are associated with reduced efficacy and increased likelihood of further interventions [12].

Suction curettage, performed as a vacuum-based uterine evacuation procedure within the framework of dilation and curettage (D&C), represents one of the surgical approaches used in the management of cervical pregnancy (CP) [16]. The procedure involves the evacuation of the gestational sac from the cervical canal, usually performed under ultrasound guidance. Due to the high risk of hemorrhage associated with this technique, it is frequently combined with other interventions to enhance safety and efficacy. For instance, balloon tamponade can be employed following suction curettage to provide hemostasis by applying pressure within the cervical canal [16]. In one study, thirteen consecutive first-trimester cervical pregnancies were successfully terminated using suction curettage followed by balloon tamponade, highlighting the effectiveness of this combined approach [16].

In recent years, hysteroscopic management has gained popularity as a fertility-preserving, minimally invasive surgical option for CP. Hysteroscopy allows direct visualization of the implantation site, precise removal of trophoblastic tissue, and the use of coagulation when needed, which may reduce the risk of massive bleeding. It may be performed as a standalone procedure or combined with systemic MTX. Several studies have reported favorable outcomes with this approach, particularly when the diagnosis is established at an early gestational age [35,36,37].

Compared with D&C, hysteroscopy appears to offer meaningful advantages in reproductive and clinical outcomes. Hysteroscopic removal of intrauterine gestational tissue allows direct visualization and targeted excision, which reduces endometrial trauma compared with “blind” curettage. Patients after a hysteroscopic procedure had a shorter time to subsequent conception and a lower risk of intrauterine adhesions and secondary infertility [38]. On the other hand, D&C remained faster and technically simpler but carried a higher risk of incomplete evacuation, mechanical injury to the endometrium and in a small number of cases even uterine perforation [39]. These findings support hysteroscopy as a fertility-preserving surgical approach, particularly in hemodynamically stable patients where a controlled, visualization-guided procedure is feasible [40].

Because severe bleeding remains one of the main therapeutic concerns in CP, additional hemostatic strategies may be required in selected patients. Uterine artery embolization (UAE) represents an adjunctive option in the management of cervical pregnancy, primarily aimed at reducing vascularity and limiting hemorrhagic complications during subsequent surgical interventions [16]. It may be particularly beneficial in advanced cases with high β-hCG levels or a large gestational sac. However, the UAE is not considered a standard first-line approach and is usually reserved for selected cases with uncontrolled bleeding or marked vascularity. Preventive UAE before surgical management has demonstrated excellent outcomes in cases with significant vascularization, high β-hCG levels and advanced gestational age, with rapid resolution of symptoms and, in many cases, preservation of fertility [21,25,30]. 

Combined or stepwise treatment strategies are frequently used in the management of CP, reflecting the clinical complexity of this condition. Systemic MTX may be combined with UAE, hysteroscopy, D&C, or cervical tamponade techniques. Additionally, mechanical compression with a Foley balloon or gauze packing has proven effective in controlling hemorrhage following curettage or evacuation. Reported cases of successful multimodal management, including combinations of multi-dose MTX, UAE, and suction curettage, further support this approach [16,21]. These multidisciplinary strategies highlight the necessity of individualized treatment plans based on clinical and ultrasonographic findings. Despite advancements, a standardized protocol for CP management remains elusive. Variations in patient presentation, clinical expertise and facility resources contribute significantly to this inconsistency.

Given the absence of a standardized treatment protocol for CP, the therapeutic approach in our patient was individualized on the basis of clinical stability, ultrasonographic findings, and the desire for fertility preservation.

### 3.4. Cesarean Scar Pregnancy Management

Cesarean scar pregnancy (CSP) represents a potentially life-threatening form of ectopic implantation, associated with substantial risk of severe hemorrhage, uterine rupture, and subsequent placenta accreta spectrum. Management of CSP remains clinically challenging and should be individualized according to gestational age, hemodynamic stability, serum β-hCG level, degree of myometrial involvement, and the patient’s reproductive wishes. Although CSP treatment is commonly divided into expectant, medical, and surgical approaches, combined or stepwise management is frequently required in clinical practice [2,41,42,43] (Table 4).

Expectant management has been reported in carefully selected cases, but it carries substantial morbidity due to progression toward placenta accreta spectrum and hemorrhagic complications, which frequently necessitate hysterectomy [54].

Non-surgical therapeutic options include local or systemic MTX administration, ultrasound-guided aspiration of the gestational sac, intra-amniotic injection of KCl, ethanol or hyperosmolar glucose, high-intensity focused ultrasound therapy (HIFU), and uterine artery embolization (UAE) [44].

Despite the availability of several conservative treatment options, a number of studies have reported the limited effectiveness of medical management alone for CSP. In particular, systemic MTX therapy has been associated with relatively low success rates and a higher probability of requiring additional interventions. Therefore, purely medical treatment should be reserved for carefully selected patients or used as part of a combined therapeutic strategy in most cases of CSP [55].

Given the limitations of medical therapy alone, surgical management has become an important component of treatment in many patients. Commonly described approaches include ultrasound-guided D&C, hysteroscopy, laparoscopic excision of the gestational sac, and in selected severe cases, laparotomy. These procedures aim to remove trophoblastic tissue while minimizing hemorrhage risk and preserving uterine integrity. Available evidence indicates the high effectiveness of surgical management. In a recent systematic review and meta-analysis, successful resolution after primary intervention was achieved in 86.2% of cases treated with ultrasound-guided suction curettage, 90.4% of those managed hysteroscopically, and 96.1% of those treated laparoscopically. Similar findings were reported in an international registry study of first-trimester CSP, further supporting the role of surgical approaches as highly effective therapeutic options. Collectively, these data indicate that surgical approaches offer high therapeutic efficacy and may be recommended as first-line treatment in appropriately selected patients with CSP [44,45,56,57].

## 4. Discussion

After a structured narrative review of PubMed/MEDLINE and Google Scholar up to March 2026, including manual screening of relevant reference lists, we did not identify a previous report describing sequential cervical pregnancy (CP) followed by cesarean scar pregnancy (CSP) in the same patient. CP and CSP are rare forms of non-tubal ectopic pregnancy characterized by implantation outside of the uterine cavity, either within the cervical canal or at the site of a previous cesarean section scar. CP accounts for less than 1% of all ectopic pregnancies, with an estimated incidence of approximately 1 in 9000–18,000 pregnancies. CSP is also uncommon, reported in approximately 1 in 1800–2500 pregnancies [8,9]. Both conditions are associated with a significant risk of severe hemorrhage and potential loss of fertility if not diagnosed and managed properly [2,10,11].

In the present case of cervical pregnancy, treatment selection was guided by the patient’s hemodynamic stability, absence of active bleeding, and preference for uterine preservation. Although conservative management appeared feasible, the presence of embryonic cardiac activity and an initial serum β-hCG level exceeding 16,000 mIU/mL were recognized as factors associated with a reduced likelihood of successful MTX monotherapy [30,33]. After shared decision-making and detailed counseling regarding available treatment options, systemic MTX was selected as the first step of a planned fertility-preserving two-step strategy intended to reduce trophoblastic activity and vascularity before delayed hysteroscopic evacuation [1,26].

Potential alternative approaches were evaluated. Primary hysteroscopic evacuation could have provided immediate tissue removal; however, because the pregnancy was viable, highly vascularized, and associated with a β-hCG level above 10,000 mIU/mL, immediate surgical intervention was considered to carry an increased risk of intraoperative hemorrhage. Local MTX or KCl injection was also considered; however, systemic MTX was selected as a less invasive initial option that could be administered promptly and did not require puncture of the highly vascular cervical implantation site. Although increased cervical vascularity was observed on Doppler imaging, prophylactic UAE was not performed during the management of CP. Considering the manageable bleeding risk in this case, and the availability of immediate hysteroscopic treatment, preventive embolization was considered unnecessary. In addition, the decision to avoid prophylactic UAE took into account its potential complications, including post-embolization syndrome, infection, ischemic injury, unintended ovarian embolization and possible effects on future reproductive function [8,9].

In this case, systemic MTX alone did not result in complete resolution of the cervical pregnancy. However, it achieved important preparatory effects: embryonic cardiac activity ceased, serum β-hCG declined, and trophoblastic vascularity was reduced. These changes allowed delayed hysteroscopic evacuation to be performed under more favorable conditions. Therefore, the combined approach should not be interpreted as successful definitive MTX monotherapy, but rather as a sequential strategy in which systemic MTX served as medical preparation for safer surgical completion.

Hysteroscopic evacuation allowed direct visualization of the cervical canal, targeted removal of residual gestational tissue, and preservation of surrounding cervical structures [23]. This combined approach enabled early, targeted intervention and avoided prolonged waiting for spontaneous abortion, which is often required when relying solely on pharmacological therapy or UAE [19,20]. In our case, complete hysteroscopic removal was achieved without excessive bleeding, and the patient remained hemodynamically stable throughout the perioperative period. Additional interventions, including uterine artery embolization, local MTX injection, KCl injection, or emergency surgery, were not required. Nevertheless, this management strategy should be interpreted with caution. Current evidence regarding combined MTX pretreatment followed by hysteroscopic evacuation for cervical pregnancy remains largely based on case reports and small case series. Further studies involving larger patient cohorts are needed to better define its effectiveness, safety, reproductive outcomes, and role in treatment algorithms before it can be recommended as a standard approach.

Equally important was contingency planning in the event of treatment failure or complications. Failure of medical therapy—commonly defined as persistently rising or plateauing β-hCG levels after MTX administration or continued viability of the gestational sac—may necessitate repeat dosing, local injection of adjunctive agents (e.g., KCl, ethanol, vasopressin), or surgical intervention such as hysteroscopic removal or suction curettage under hemostatic control [58]. In cases of uncontrolled hemorrhage, emergency UAE or hysterectomy may be life-saving. These measures are generally reserved for severe or refractory cases and are not part of routine management [32,59]. It is important to emphasize that individualized treatment should always incorporate predefined backup strategies to balance fertility preservation with patient safety.

Several patient-related factors may have increased the complexity of management in this case, including previous uterine surgery, three prior cesarean deliveries, and ongoing smoking. These factors may contribute to altered uterine architecture, abnormal implantation, and potentially reduced response to medical treatment alone [7,60]. In this context, the case illustrates the importance of individualized treatment planning, careful assessment of hemorrhagic risk, and anticipation of the possible need for combined or stepwise management. Although the favorable clinical course supports the feasibility of a fertility-preserving approach in carefully selected high-risk patients, this observation should be interpreted cautiously, given the limited evidence base and the single-case nature of the report.

An important limitation of this case was the incomplete follow-up after postoperative day 23. At the scheduled follow-up visit, serum β-hCG had declined substantially to 65.74 mIU/mL and liver function tests were within normal limits. However, no further serial β-hCG measurements were obtained at that time, as the patient did not attend subsequent planned follow-up visits because of the long distance from our center. Approximately five weeks after the procedure, she was contacted by telephone and reported no symptoms such as pain, bleeding, or fever. Although her clinical well-being was reassuring, the absence of serial biochemical monitoring during this period prevents absolute certainty regarding the exact time to complete resolution and does not allow persistent trophoblastic tissue to be definitively excluded during the follow-up gap.

Six months after treatment, the patient presented for an in-person follow-up visit. At that time, serum β-hCG was negative, and transvaginal ultrasound demonstrated normal uterine and cervical anatomy, supporting complete resolution of the cervical pregnancy and confirming a favorable final clinical outcome. Nevertheless, assessment of long-term reproductive outcomes remains limited. Although the patient conceived again approximately one year later, confirming subsequent fertility, this pregnancy was complicated by cesarean scar implantation and therefore does not provide evidence of an uncomplicated reproductive outcome after cervical pregnancy treatment. Data regarding subsequent normally implanted intrauterine pregnancy, live birth, cervical competence, or other obstetric outcomes were not available. Therefore, although uterine preservation was achieved and subsequent conception occurred, this report cannot determine the effect of the treatment strategy on future uncomplicated fertility or obstetric outcomes.

One year after CP treatment, the patient presented again with suspected early pregnancy. Diagnostic evaluation confirmed implantation within the previous cesarean section scar, representing an extremely rare sequential occurrence of two distinct non-tubal ectopic pregnancies in the same patient.

In the present case of cesarean scar pregnancy (CSP), hysteroscopic evacuation was selected. Given the early gestational age, absence of fetal cardiac activity, and hemodynamic stability, a minimally invasive procedure was considered an appropriate treatment option. Direct visualization of the implantation site within the cesarean scar allowed precise removal of the trophoblastic tissue while limiting injury to the surrounding myometrium and reducing the risk of uncontrolled bleeding. This approach enabled definitive treatment in a single procedure, while avoiding more extensive surgery and preserving uterine integrity. The uncomplicated perioperative course and complete evacuation of the gestational tissue observed in our patient further support the use of hysteroscopic management in appropriately selected cases of CSP. One month after the procedure, serum β-hCG was negative, confirming complete resolution of the pregnancy.

Finally, the reproductive implications of the fertility-preserving management used in this case remain uncertain. Although conservative procedures allowed anatomical preservation of the uterus, this does not necessarily demonstrate maintenance of normal reproductive function. As the subsequent conception was complicated by abnormal implantation, and no normally implanted intrauterine pregnancy or live birth was documented, this report cannot determine whether the fertility-preserving approach was associated with a favorable obstetric outcome.

## 5. Conclusions

This case highlights the exceptional rarity of the sequential occurrence of two distinct non-tubal ectopic pregnancies in the same patient. It emphasizes the importance of early transvaginal ultrasonography, timely diagnosis, and individualized multidisciplinary management in women with risk factors for abnormal implantation. This case suggests that, in carefully selected hemodynamically stable patients with cervical pregnancy who wish to preserve fertility, a two-step strategy consisting of systemic methotrexate followed by delayed cold-loop hysteroscopic evacuation may enable effective fertility-preserving treatment, even when initial clinical features suggest an increased risk of methotrexate failure. Hysteroscopic management may also represent a minimally invasive option in selected cases of cesarean scar pregnancy in patients wishing to preserve reproductive potential. Structured post-treatment follow-up with serial serum β-hCG measurements remains essential to confirm complete resolution and detect persistent trophoblastic tissue or treatment failure.

## Figures and Tables

**Table 1:** Classification of ectopic pregnancy types.

EP Type	Description	Incidence
Tubal	Gestational sac implant in the fallopian tube (most commonly in tubal ampulla).	95%
Interstitial	Gestational sac implants in interstitial portion of the fallopian tube.	2–4%
Cesarean scar	Gestational sac implants into the anterior uterine wall of lower uterine segment where cesarean scar resides.	<1%
Cervical	Gestational sac implants in the mucosa of the endocervical canal.	<1%
Ovarian	Gestational sac implants in the ovaries.	<3%
Abdominal	Gestational sac implants in the peritoneal cavity of the abdomen.	1%

**Table 2:** Evolution of serum β-hCG and presence of fetal heart rate (FHR) during methotrexate treatment of cervical pregnancy. MTX—methotrexate; HSC—hysteroscopy; FHR (+)—fetal heart rate present; FHR (−)—fetal heart rate absent.

Day of Hospitalization	Intervention	B-HCG [mIU/mL]	FHR
1	MTX 92 mg	16,818	+
2	−	−	+
3	−	−	+
4	MTX 92 mg	23,805	+
5	−	27,867	+
6	−	29,600	+
7	−	23,549	−
8	−	23,449	−
9	−	−	−
10	−	17,126	−
11	HSC	−	−
23	−	65.74	−

**Table 3:** Reported effectiveness, safety profile, and reproductive outcomes of treatment options for cervical pregnancy. Quality of evidence was assessed according to the type and consistency of published data supporting each treatment modality. High quality indicates evidence derived from larger comparative studies or systematic reviews; moderate quality indicates evidence from retrospective studies or multiple case series; low quality indicates evidence based primarily on case reports or small case series. Given the rarity of cervical pregnancy, most data are derived from retrospective studies, case studies, case series, and case reports. MTX—methotrexate; D&C—dilatation and curettage; HSC—hysteroscopy; UAE—uterine artery embolization [10,11,12,13,14,15,16,17,18,19,20,21,22,23,24].

Modality	Effectiveness	Safety Profile	Reproductive Outcomes	Quality of Evidence	Key Supporting Articles
Intra-amniotic MTX injection	73.3% with a single local injection	Low reported rate of major complications; additional treatment may be required. 81% uncomplicated cases. No complications in 81% of locally treated cases in the Ferrara literature review; transfusion and infection each 3%; hysterectomy 1 case	Favorable, with high likelihood of uterine preservation and good future fertility potential in successfully treated patients.	Low–moderate	Yamaguchi et al., 2017; Ferrara et al. [10,11]
Systemic MTX	~91%	Effective in hemodynamically stable patients with early cervical pregnancy, but hemorrhage, treatment failure, and need for additional procedures may occur. Medical management is often combined with other conservative measures in selected cases.	Generally favorable, with preservation of fertility in most successfully treated patients.	Moderate	Kung and Chang, 1999; Kirk et al., 2006; Murji et al., 2015 [12,13,14]
D&C	79.2% overall successful outcome without major complications or re-intervention	Less favorable as a stand-alone procedure because of severe hemorrhage risk and uterine rupture. Safer when combined with adjunctive hemostatic measures such as balloon tamponade or UAE	Variable; fertility may be preserved if bleeding is controlled, but the hemorrhage risk makes outcomes less favorable.	Low–moderate	He et al., 2026; Fylstra, 2014; Fowler et al., 2021 [15,16,17]
HSC	100% success in small selected series	No major complications reported in those small selected hysteroscopy series; broader CP review literature describes hysteroscopy as feasible and safe, especially in early cases	Generally favorable; hysteroscopy is presented as a fertility-sparing, uterine-preserving approach.	Low	Maglic et al., 2021; Tanos et al., 2019; Di Lorenzo et al., 2022 [18,19,20]
UAE + D&C	97.7% overall successful outcome without major complications or re-intervention	Lower blood loss and improved perioperative safety compared with D&C alone, but associated with post-embolization syndrome and delayed menstrual recovery.	Generally favorable.	Moderate	He et al., 2026; Hu et al., 2016; Wang et al., 2011; Hirakawa et al., 2009 [15,21,22,25]
UAE + HSC	~100% (reported in small series)	Favorable; negligible blood loss, no transfusion, no conversion, no reported complication	Fertility-sparing	Low	Scutiero et al., 2013; Vilos et al., 2005; Di Lorenzo et al., 2022 [20,23,24]

**Table 4:** Reported effectiveness, safety profile, and reproductive outcomes of treatment options for cesarean scar pregnancy. Quality of evidence was assessed according to the type and consistency of published data supporting each treatment modality. High quality indicates evidence derived from larger comparative studies or systematic reviews; moderate quality indicates evidence from retrospective studies or multiple case series; low quality indicates evidence based primarily on case reports or small case series. Given the rarity of cesarean scar pregnancy, most data are derived from retrospective studies, case studies, case series, and case reports. CSP—cesarean scar pregnancy; MTX—methotrexate; D&C—dilatation and curettage; HSC—hysteroscopy; UAE—uterine artery embolization; HIFU—high-intensity focused ultrasound.

Modality	Effectiveness	Safety Profile	Reproductive Outcomes	Quality of Evidence	Key Supporting Articles
Intra-amniotic MTX injection	81.6% pooled success.	5.9% pooled complication rate. Risk of persistent tissue, reintervention, and hemorrhage.	Fertility-preserving in principle, but direct long-term data are limited.	Low–moderate	Alameddine et al., 2024; Agten et al., 2024; SMFM Consult Series #63, 2022 [43,44,45].
Systemic MTX	59.4% in the international registry; pooled meta-analytic estimate 72.4%	3.5% pooled post-treatment complication rate. High failure and reintervention risk.	Fertility-preserving in principle, but direct long-term data are limited.	Low	Agten et al., 2024; Alameddine et al., 2024; SMFM Consult Series #63, 2022 [43,44,45].
D&C	86.2% pooled success; 91.5% in the international registry.	Generally favorable under ultrasound guidance, but associated with a risk of bleeding, persistent tissue, and reintervention; sharp curettage alone should be avoided. Pooled post-treatment complications were reported at 5.5%.	Future pregnancy is achievable; in one 2024 BMC cohort, overall reproductive outcomes among women desiring fertility included 43% live birth and 15% recurrent CSP.	Low–moderate	Alameddine et al., 2024; Agten et al., 2024; SMFM Consult Series #63, 2022; Lei et al., 2024. [43,44,45,46].
HSC	90.4% pooled success.	1.4% pooled complication rate; hemorrhage occurred in 1.66% and hysterectomy in 0.28% of cases	Subsequent pregnancy is achievable; in the Tang et al. follow-up study, 37 women conceived again, corresponding to 48.7% (37/76) of those with available follow-up, 22 women achieved term pregnancy.	Low–moderate	Alameddine et al., 2024; Fu et al., 2024; Diakosavvas et al., 2022; Tang et al., 2021. [44,47,48,49]
Laparoscopy	96.1% pooled success.	Generally favorable; no post-treatment complications were reported in pooled meta-analytic data, and network meta-analysis ranked laparoscopy among the safer options. It also allows simultaneous excision and scar repair, which may be advantageous in selected patients.	Potentially favorable because scar repair may reduce recurrence risk of CSP.	Low–moderate	Alameddine et al., 2024; Fu et al., 2024; SMFM Consult Series #63, 2022. [43,44,47]
HIFU + D&C	98.3% reported success; the network meta-analysis ranked HIFU + suction curettage among the top-performing options.	Pooled post-treatment complications were 13.9%; most reported adverse effects were transient pain-related symptoms or fever, while major complications were uncommon in cohort studies.	Subsequent pregnancy appears favorable; reported follow-up data show a 68.7% subsequent pregnancy rate, and one series found that 82.1% (23/28) of women desiring pregnancy conceived after treatment.	Low–moderate	Fu et al., 2024; Alameddine et al., 2024; Yung et al., 2024; Jiang et al., 2024; Zhang et al., 2019. [43,47,50,51,52]
UAE + D&C	91.9% pooled success.	13.9% pooled complication rate; complications may include post-procedural pain, fever, and bleeding-related events.	Limited evidence from a small retrospective follow-up study suggests favorable reproductive outcomes after UAE + D&C, with 82.6% subsequent conception, 78.9% live birth, and 5.3% recurrent CSP.	Low	Alameddine et al., 2024; Fu et al., 2024; Chen et al., 2022 [44,47,53].
UAE + HSC	91.1% pooled success.	16.6% pooled post-treatment complication rate.	Fertility-preserving in principle, but modality-specific long-term reproductive outcome data for UAE + HSC remain limited.	Low	Alameddine et al., 2024; Fu et al., 2024 [44,47].

## Data Availability

The data supporting the findings of this case report are not publicly available due to patient privacy considerations but are available from the corresponding author on reasonable request.

## Abbreviations

The following abbreviations are used in this manuscript:

ALT	alanine aminotransferase
ART	assisted reproductive technologies
AST	aspartate aminotransferase
CP	cervical pregnancy
CRL	crown-rump length
CSP	cesarean scar pregnancy
D&C	dilatation and curettage
EP	ectopic pregnancy
FHR	fetal heart rate
HIFU	high-intensity focused ultrasound
HSC	hysteroscopy
IUD	intrauterine device
KCl	potassium chloride
MTX	methotrexate
SMFM	Society for Maternal-Fetal Medicine
UAE	uterine artery embolization
β-hCG	beta-human chorionic gonadotropin
